# Selenite Reduction by Anaerobic Microbial Aggregates: Microbial Community Structure, and Proteins Associated to the Produced Selenium Spheres

**DOI:** 10.3389/fmicb.2016.00571

**Published:** 2016-04-26

**Authors:** Graciela Gonzalez-Gil, Piet N. L. Lens, Pascal E. Saikaly

**Affiliations:** ^1^Division of Biological and Environmental Sciences and Engineering, Water Desalination and Reuse Center, King Abdullah University of Science and TechnologyThuwal, Saudi Arabia; ^2^Department of Environmental Engineering and Water Technology, UNESCO-IHE Institute for Water EducationDelft, Netherlands

**Keywords:** selenium, anaerobic granules, microbial aggregates, granular sludge, nanoparticles, nanospheres, biomineralization, bioreduction

## Abstract

Certain types of anaerobic granular sludge, which consists of microbial aggregates, can reduce selenium oxyanions. To envisage strategies for removing those oxyanions from wastewater and recovering the produced elemental selenium (Se^0^), insights into the microbial community structure and synthesis of Se^0^ within these microbial aggregates are required. High-throughput sequencing showed that *Veillonellaceae* (c.a. 20%) and *Pseudomonadaceae* (c.a.10%) were the most abundant microbial phylotypes in selenite reducing microbial aggregates. The majority of the *Pseudomonadaceae* sequences were affiliated to the genus *Pseudomonas*. A distinct outer layer (∼200 μm) of selenium deposits indicated that bioreduction occurred in the outer zone of the microbial aggregates. In that outer layer, SEM analysis showed abundant intracellular and extracellular Se^0^ (nano)spheres, with some cells having high numbers of intracellular Se^0^ spheres. Electron tomography showed that microbial cells can harbor a single large intracellular sphere that stretches the cell body. The Se^0^ spheres produced by the microorganisms were capped with organic material. X-ray photoelectron spectroscopy (XPS) analysis of extracted Se^0^ spheres, combined with a mathematical approach to analyzing XPS spectra from biological origin, indicated that proteins and lipids were components of the capping material associated to the Se^0^ spheres. The most abundant proteins associated to the spheres were identified by proteomic analysis. Most of the proteins or peptide sequences capping the Se^0^ spheres were identified as periplasmic outer membrane porins and as the cytoplasmic elongation factor Tu protein, suggesting an intracellular formation of the Se^0^ spheres. In view of these and previous findings, a schematic model for the synthesis of Se^0^ spheres by the microorganisms inhabiting the granular sludge is proposed.

## Introduction

Phylogenetically diverse microorganisms are able to reduce the soluble forms of selenium selenate (SeO_4_^2-^) and selenite (SeO_3_^2-^) into insoluble elemental selenium (Se^0^) (Stolz et al., 2006; Pearce et al., 2009). This biomineralization occurs when SeO_4_^2^ (Macy and Lawson, 1993; Oremland et al., 1994; Switzer Blum et al., 2001) or SeO_3_^2-^ (Switzer Blum et al., 1998; Baesman et al., 2009) are utilized as respiratory electron acceptors by some anaerobic bacterial species to support growth, or via a detoxification mechanism in which bioreduction is not coupled to growth but occurs as response to cope with the toxicity of the selenium oxyanions (Tomei et al., 1995; Kessi et al., 1999). Among the few species of SeO_4_^2-^ respiring bacteria reported thus far, *Thauera selenatis* is the most studied and its periplasmic selenate reductase complex has been investigated extensively (Schröder et al., 1997; Stolz et al., 2006). Conversely, only two species of SeO_3_^2-^ respiring bacteria have been isolated until now and details regarding the bioreduction are not fully understood (Switzer Blum et al., 1998; Stolz et al., 2006). Either through anaerobic respiration or detoxification, selenium is mainly reduced to Se^0^ spherical particles of sizes ranging from nano to submicron scales of about 20–500 nm (Oremland et al., 2004; Pearce et al., 2009). Given the wide range of sphere sizes and that some can fall out of the definition of nanoparticles (i.e., nano < 100 nm in at least one dimension; Horie et al., 2012), the term Se^0^ spheres is used throughout in this manuscript.

The selenium bioreduction mechanism has attracted attention to be used for remediating sites and treating wastewaters contaminated with selenium oxyanions (Chung et al., 2006; Williams et al., 2013). The use of biological methods to treat selenium containing waste waters is preferred over physicochemical methods because the removal process can proceed under mild conditions without the generation of hazardous residues or the use of costly reagents (Astratinei et al., 2006; Lenz and Lens, 2009). Considering the acute toxicity of selenium oxyanions, industries such as mining, coal combustion, oil refinery, and glass and electronics production (Lemly, 2004; Maher et al., 2010) are currently challenged to reduce selenium concentrations in their eﬄuents (e.g., <5 μg/l) (EPA, 2009).

Wastewater treatment systems for selenium oxyanion bioreduction have been developed using pure cultures of *T. selenatis* (Cantafio et al., 1996; Bledsoe et al., 1999). However, the use of pure cultures to treat wastewaters in the long term is logistically not recommended as wastewaters are not sterile. Previous studies showed that anaerobic granular sludge –quasi-spherical aggregates of multispecies microbial consortia– is able to reduce SeO_4_^2-^ and SeO_3_^2-^ at concentrations ranging from 0.01 to 1 mM with removal efficiencies from 36 to 90%, depending on the initial concentrations and source of biomass (Astratinei et al., 2006; Lenz et al., 2008a, 2011b). Most of the reduced selenium in the granular sludge was found as Se^0^ (Lenz et al., 2011b), thus the use of anaerobic granular sludge for selenium bio-reduction in bioreactors appears very attractive. However, previous research showed that not all, but only certain types of granular sludge could reduce selenium oxyanions (Astratinei et al., 2006). It is thus of great relevance to investigate which microbial community are present in a suitable granular sludge and whether (or how) the microbial community changes when the granular sludge is exposed to selenium oxyanion containing wastewater.

Considering the increased interest in the use of selenium in high tech applications (Dou et al., 2013), treatment systems should not only aim at removing dissolved selenium, but should also consider recovering strategies of valuable Se^0^ spheres. Thus, for recovering purposes, it is relevant to investigate the localization of the formed Se^0^ spheres at the granular sludge- and cell-scale. These features have not yet been investigated in anaerobic microbial aggregates (i.e., anaerobic granular sludge). Research using pure cultures of bacteria isolated mainly from selenium-contaminated sites show that Se^0^ spheres are found both intracellularly and extracellularly (Losi and Frankenberger, 1997; Kessi et al., 1999; Oremland et al., 2004). However, the mechanisms of biosynthesis of Se^0^ spheres and their export from the cell, if present therein, are not well elucidated. It is proposed that identifying the proteins associated to the Se^0^ spheres may contribute to understanding the mechanisms of their biosynthesis (Debieux et al., 2011).

Few studies have investigated the proteins associated to Se^0^ spheres (Dobias et al., 2011; Lenz et al., 2011a) and only qualitative information was obtained, but the involvement of certain proteins in the bioreduction process may be better understood if (semi)quantitative proteomic information is available. Therefore, the aims of this study were (i) to determine which microbial types are present in a suitable inoculum and how the community develops when the sludge is fed with selenite, (ii) to assess, at the aggregate- and cell-scales, the localization of the formation of Se^0^ spheres by anaerobic granular sludge, and (iii) to identify and semi-quantify proteins associated to the Se^0^ spheres produced by the anaerobic granular sludge. To address these objectives, a combination of electron microscopic, high-throughput pyrosequencing and proteomic approaches were employed. In view of the findings, along with knowledge from previous research, a schematic model for the bacterial synthesis of Se^0^ spheres inhabiting the granular sludge was proposed.

## Materials and Methods

### Source of Se^0^ Containing Biomass

The inoculum anaerobic granular sludge originated from a full-scale reactor treating brewery wastewater, information regarding operational conditions of this reactor was reported previously (Gonzalez-Gil et al., 2001). The sludge (13.5 g/L wet sludge) was incubated in a 500 ml batch reactor with anaerobic mineral medium (pH = 7.3) containing the following components: 5.6 mM NH_4_Cl, 0.1 mM CaCl_2_.2H_2_O, 1.8 mM KH_2_PO_4_, 2.0 mM Na_2_HPO_4_, and 3.3. mM KCl. Sodium lactate was added as electron donor at 20 mM. Selenite was added as Na_2_SeO_3_ at 5 mM. The reactor was flushed with N_2_ and then incubated for 21 days at 30°C. Under these conditions and with similar anaerobic granular sludge, preliminary assays showed that more than 90% of selenite is reduced to Se^0^, and no Se^0^ formation occurred without lactate addition or with killed biomass as detailed previously (Astratinei et al., 2006; Lenz et al., 2011b). Furthermore, at this experimental stage, batch systems were utilized to avoid potential heterogeneity of granular sludge resulting from plug-flow hydraulic regimes associated with the operation of sludge bed up-flow reactors (Wu and Hickey, 1997).

### Recovery of Se^0^ Spheres

After incubation of anaerobic sludge with selenite, solids were recovered by centrifugation (37,000 ×*g*, 30 min at 4°C). The pellet was re-suspended in ultrapure water (18 M Ohm cm) and the Se^0^ spheres were separated from the biomass using the procedure of Dobias et al. (2011) with some modifications as previously described (Jain et al., 2015). Briefly, Se^0^ spheres were extracted from biomass using sonication followed by an alkaline treatment. The spheres were then separated from biomass debris in a separatory funnel using n-hexane. The organic phase retained the biomass and the Se^0^ spheres were then recovered from the aqueous phase. The recovered particles were washed and stored under anaerobic conditions.

### DNA Extraction, Amplification and 454 Pyrosequencing of 16S rRNA Gene Amplicons

Genomic DNA was extracted from known amounts of the granular sludge before (i.e., inoculum granules) and after incubation with selenite (i.e., selenite reducing granules) using the Power Soil DNA isolation kit (MoBio, Inc.) as described in detail previously (Gonzalez-Gil et al., 2015). Bacterial and archaeal 16S rRNA gene fragments were amplified as described before (Gonzalez-Gil et al., 2015). The amplified 16S rRNA gene fragments were pyrosequenced using a Roche 454 GS FLX sequencer and Titanium reagents according to the manufacturer’s protocols.

### Analysis of 16S rRNA Gene Pyrosequencing Data

The obtained sequences were analyzed using the QIIME pipeline^1^ (Caporaso et al., 2010). Briefly, sequences were quality filtered (i.e., length ≥200 bp, sequence quality ≥25, no ambiguous bases and homopolymer runs length <6), and primers and adapters were trimmed. Sequences were denoised, clustered at 97% sequence similarity into operational taxonomic units (OTUs) and chimeras were removed. Final OTUs were aligned using PyNAST and taxonomically classified using the RDP classifier and the Greengenes database. Further details can be found in (Gonzalez-Gil et al., 2015). After quality filtered, a total of 20887 and 18787 reads (Table S1) were obtained for the inoculum and selenite reducing granules, respectively. Pyrosequencing reads were deposited in the NCBI Short-Read Archive under BioProject number PRJNA280561 with bacteria reads accession number SRR2175653 and archaea reads accession number SRR2175722.

### Quantitative PCR

Quantitative PCR (qPCR) was conducted using a CFX96 real-time detection apparatus (Bio-Rad Laboratories, Hercules, CA, USA). Bacterial and archaeal 16S rRNA gene fragments were amplified using the same primer pairs as those used for the pyrosequencing analysis. Details of the primer pairs and reaction conditions can be found in the supporting information. To quantify copy numbers of bacterial and archaeal 16S rRNA genes, standard curves (ranging from 2 × 10^4^ to 2 × 10^8^) for bacteria (slope = -3.7, *r*^2^= 0.996, efficiency = 86%) and archaea (slope = -3.8, *r*^2^= 0.994, efficiency = 83 %) were constructed for each qPCR run by using dilution series of known concentrations of purified bacteria and archaea amplicons amplified from genomic DNA from *Geobacter sulfurreducens* and *Methanosarcina barkeri*, respectively.

The total (i.e., net) abundance of different phylotypes were estimated as follows:

$${TA}_{p}=C_{k}\times F_{p}$$

where, TA_P_ is the total abundance of a certain bacterial or archaeal phylotype (16S rRNA gene copy number per gram of sludge dry weight), C_K_ is the abundance of bacteria or archaea estimated by qPCR (16S rRNA gene copy number per gram of sludge dry weight), and F_P_ is the fraction of a phylotype obtained from the bacterial or archaeal 16S rRNA gene pyrosequencing analysis.

### Electron Microscopy and Tomography

Electron microscopy was conducted as detailed previously (Gonzalez-Gil et al., 2015). Briefly, granules were fixed in 2.5% glutaraldehyde in phosphate buffer saline pH 7.2, stored at 4°C overnight and washed with water to remove the fixative. For TEM analysis, a drop of sludge suspension was placed on a Cu grid. After staining with uranyl acetate 1% in water, samples were imaged using a Titan 80–300 electron microscope (Titan Cryo Twin; FEI Company) operating at 300 kV. For tomographic reconstruction, series of tilt images were captured using a Saxton scheme (Koning and Koster, 2013) at 2° intervals in the tilt range of -68° to 68° using the Xplore 3D tomography software (FEI company). The tomogram was generated using the Weighted-Back Projection algorithm using the IMOD software (Kremer et al., 1996). Segmentation and 3D rendering of tomographic images was done using the image-processing software Avizo (Visualization Science Group). For SEM analysis, fixed granules (as described above) were cross-sectioned and then freeze dried with slush N_2_. Samples were then coated with carbon (5–10 nm) and observed using a Quanta 3D FEG electron microscope (FEI, The Netherlands). Energy dispersive X-ray (EDX) elemental mapping was done with an accelerating voltage of 25 kV.

### X-ray Photoelectron Spectroscopy (XPS)

X-ray photoelectron spectroscopy spectra of Se^0^ spheres were acquired with a Kratos Axis Ultra DLD spectrometer using a monochromatic Al Kα X-ray source (hα = 1486.6 eV) operated at 150 W and with a multi-channel plate and delay line detector under 1.0 × 10^-9^ Torr vacuum. The survey and high-resolution spectra were collected at fixed analyzer pass energies of 160 and 20 eV, respectively. The spectrometer charge neutralizing system was used to compensate for sampling charging during measurements. Binding energies were referenced to the C 1s aliphatic carbon peak at 285.0 eV and the average of six spectra was used for further analysis. Peak deconvolution was done using XPSPEAK v4.1. The proportion of biological components contributing to the C 1s signal was estimated using the compfit.m routine (Ramstedt et al., 2011) run in Matlab.

### Proteomic Analysis of Se^0^ Spheres Associated Proteins

Proteins associated to the Se^0^ spheres were extracted from biological replicates, purified and digested with tripsin based on the protocol of Thomas et al. (2013) (see details in the Supporting Information). Digested peptide mixtures were resuspended in 5% (v/v) acetonitrile (ACN) and 0.1% (v/v) formic acid and analyzed on an LTQ Orbitrap Velos mass spectrometer (MS; Thermo Scientific, Bremen, Germany) as described by Thomas et al. (2013), except that the flow of the mobile phase was 400 nL min^-1^. The obtained spectra were submitted to a local MASCOT (version 2.4.0; Matrix Science, London, UK) server and set-up to search the Swiss-Prot database (release 2012). The procedure for the identification of proteins can be found in the Supporting Information.

## Results

### Microbial Community Composition of the Anaerobic Granules before and after Exposure to Selenite

**FIGURE 1:**
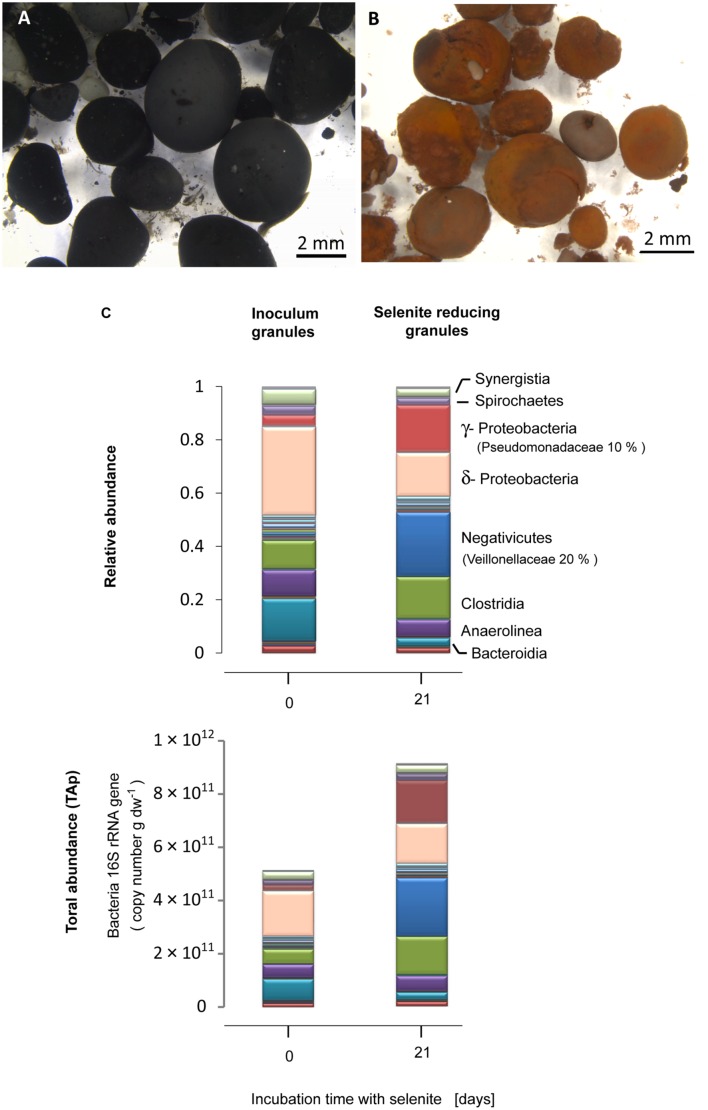
**Anaerobic granular sludge (A) before and (B) after 21 days incubation with selenite. (C)** Bacterial community composition expressed in relative abundance and total abundance of phylotypes (TA_P_) (16S rRNA gene copy number), at the class level, of the inoculum anaerobic granules and the selenite reducing granules. The families of most abundant microorganisms in the selenite reducing granules are given in parenthesis.

The black granular sludge used as inoculum acquired a reddish coloration characteristic of amorphous elemental selenium during the incubation with selenite (**Figures 1A,B**). The inoculum contained δ-*Proteobacteria* (33%) as the most abundant bacterial class, followed by *Bacteroidia* (15%), *Clostridia* (10%) and *Anaerolinea* (10%) (**Figure 1C**). Exposure of the granules to selenite for 21 days induced a change in the microbial community structure. In the selenite reducing granules, γ-*Proteobacteria* (17%) and *Negativicutes* (24%) developed as the dominant bacterial classes from which the families *Pseudomonadacea* (10%) and *Veillionelaceae* (20%) were the most abundant, respectively (**Figure 1C**). About 90% of the *Pseudomonadaceae* were affiliated to the genus *Pseudomonas* (Table S2, tab genus level). The *Veillionellacea* sequences could only be classified at the family level (Table S2). Further phylogenetic details for the phylotypes of the inoculum and the selenite reducing granules can be found in the Supporting Information (Figure S1; Table S2).

The qPCR results showed that, with respect to the inoculum, there was a significant increase in the bacterial population in the selenite reducing granules (*t*-test, *P* = 0.05) (Figure S2A). Combining bacterial qPCR results (Figure S2) with pyrosequencing reads allowed to examine which bacterial taxa exhibited a net abundance increase following selenite exposure. An increase in the TA_P_ of γ-*Proteobacteria*, *Negativicutes*, and *Clostridia* was observed in the selenite reducing granules in comparison to the inoculum granules (**Figure 1C**).

Quantitative PCR analysis showed that archaeal 16S rRNA gene copy numbers per gram dried weight of granular sludge did not change significantly after the granular sludge was exposed to selenite (*t*-test, *P* = 0.57) (Figure S2A).

### Macro- and Micro-Scale Localization of the Bioreduction Process

**FIGURE 2:**
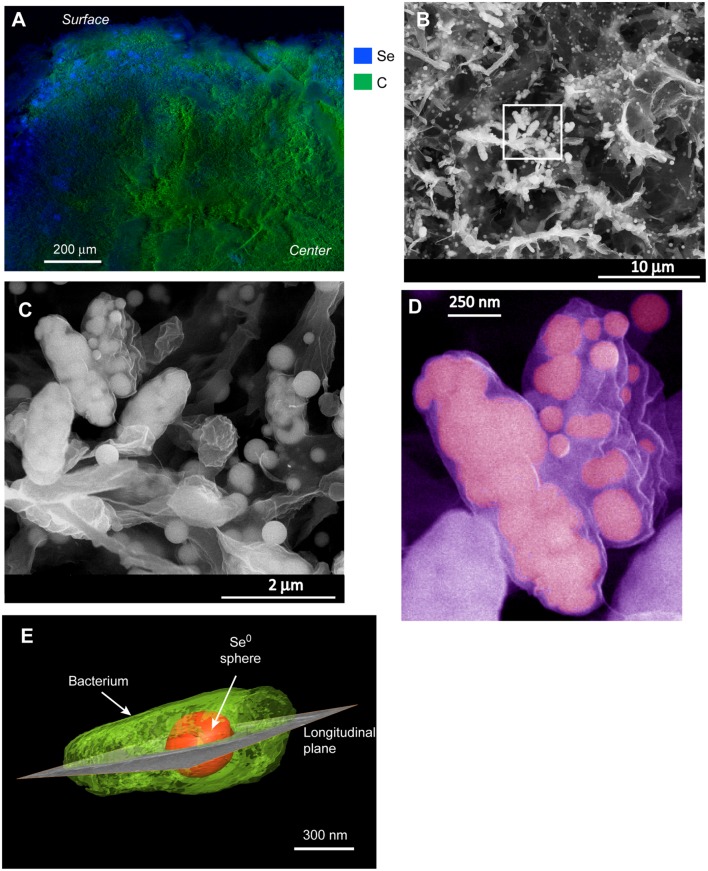
**(A)** EDX analysis of a cross-sectioned selenite reducing granule showing that selenium deposition mainly occurred on the surface of the granule. **(B)** SEM image from a blue area in **(A)** showing a mixture of microbial cells and selenium (Se^0^) particles. **(C)** zoom-in image from the boxed area in **(B)** showing Se^0^ spheres apparently inside cells as well as extracellular Se^0^ spheres. **(D)** Cells in **(C)** showing details of internal spheres; the image was pseudo-colored. **(E)** 3-D reconstruction of a microbial cell having an intracellular Se^0^ sphere.

Energy dispersive X-ray analysis of cross-sectioned granules showed that Se was mostly localized in the outer ∼100–200 μm part of the granules (**Figure 2A**). Few discrete (patchy) EDX signals of selenium were observed in the interior of the granule, suggesting that although selenite could probably well diffuse and reach the interior, most microorganisms producing Se^0^ were localized in the outer part of the granules. In the outer zone, high magnification SEM images showed abundant deposition of Se^0^ spheres (**Figure 2B**), which appeared both inside and/or outside microbial cells (**Figures 2B,C**). Some cells appeared having large numbers of internal Se^0^ spheres (**Figure 2D**). Since conventional TEM images provide only 2D information, electron tomography was used to overcome this limitation. Using TEM tomography, a 3D reconstruction of a sample revealed the intracellular localization of a fairly large Se^0^ sphere in the investigated cell (**Figure 2E**).

**FIGURE 3:**
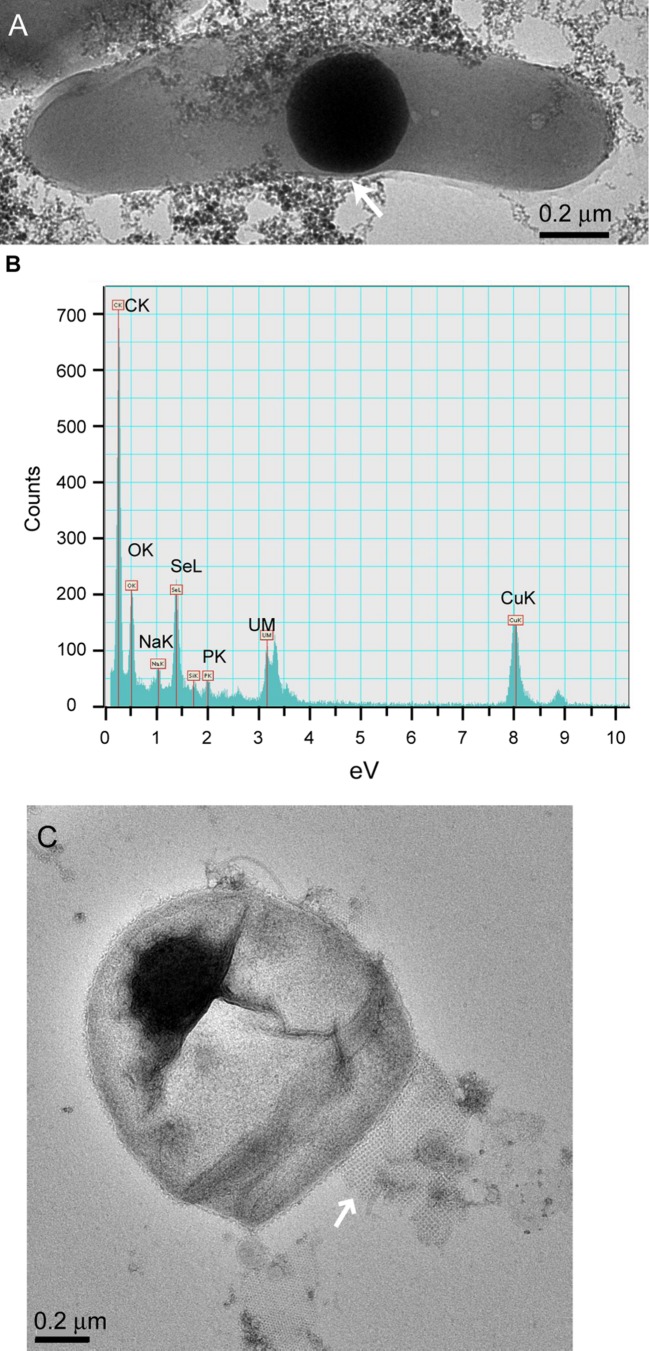
**(A)** TEM image of dispersed samples of anaerobic granular sludge exposed to selenite showing a microbial cell and intracellular Se^0^ sphere. “Stretching” of the outer membrane is indicated by the arrow. **(B)** EDX analysis of the area on **(A)** showing Se signal. The copper signal is due to the grid used to support the sample. **(C)** Se^0^ particle on the edge of a fractured cell. A remain of the S-layer, with apparently tetragonal (p4) symmetry (Sleytr and Beveridge, 1999; Pavkov-Keller et al., 2011) is visible (arrow).

Cells in the granular sludge produced intracellularly multiple (**Figure 2D**) or single (**Figure 2E**) Se^0^ spheres. A single intracellular sphere could, proportionally to the cell, be so large (occupying about one third of the cell volume) that the cell body appeared to be stretched to accommodate the particle (**Figure 3A**). A selenium signal was clearly detected by EDX analysis of that particle (**Figure 3B**). Strained imposed by a (internal) sphere might induce fracture of the microbial cell as shown in **Figure 3C**. When Se^0^ spheres were not observed intracellularly, they were nearby lysed cells (Figure S3). Cell lysis due to preparation procedures is unlikely since intact cells were also observed, and these intact cells did not show Se^0^ particles (Figure S4). Furthermore, the S-layer, paracrystalline lattice of self-assembled proteinaceous subunits (Sleytr and Beveridge, 1999; Pavkov-Keller et al., 2011), was visible in some of those intact cells (Figure S4C), while remains of the S-layer were observed in damaged cells (**Figure 3C**).

### High Magnification Imaging of the Se^0^ Spheres

**FIGURE 4:**
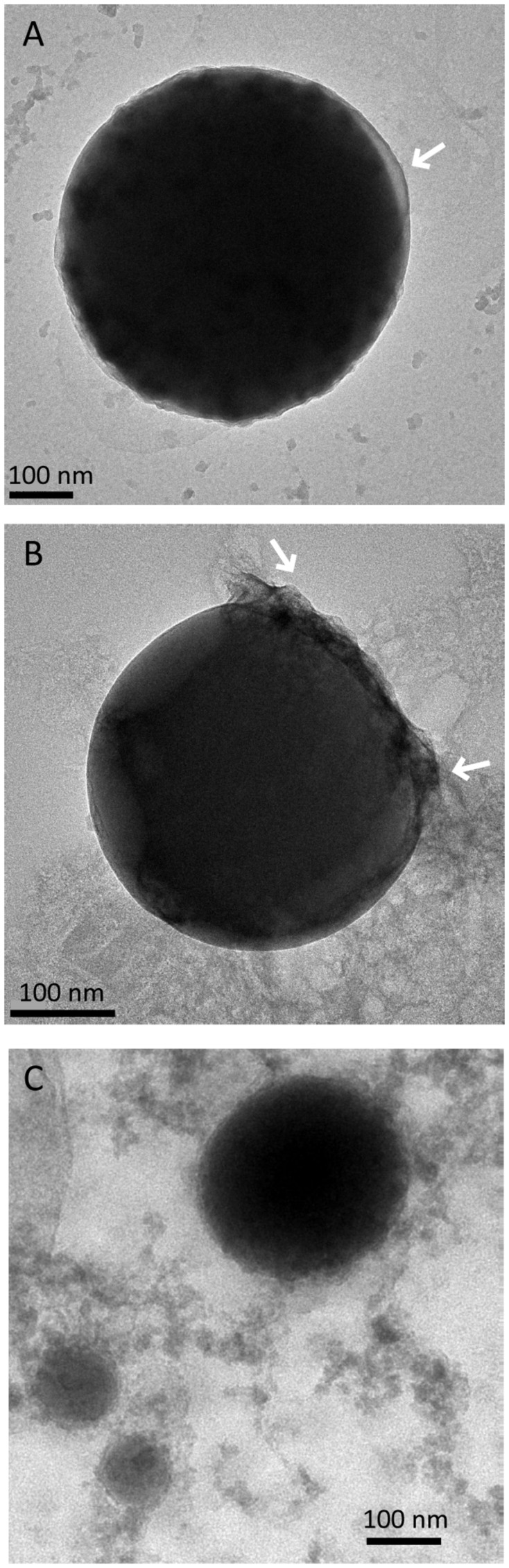
**Selenium spheres produced by the anaerobic granules. (A)** Arrows indicate non-selenium material attached to the spheres. **(B)** The two arrows indicate a face of attachment. **(C)** Se^0^ nanoparticles coalescing or aggregating to form larger Se^0^ spheres.

The Se^0^ spheres produced by the granular sludge were spherical with an average diameter of 313 (±81) nm (*n* = 130) and more than 75% of the spheres were larger than 260 nm (Figure S5). When observed using high magnification TEM, pellicle-like structures appeared attached or associated to the Se^0^ spheres (**Figure 4A**). On a Se^0^ particle, a plane of attachment was observed (**Figure 4B**). Dedicated kinetic studies are required to track the nucleation and growth of the Se^0^ spheres, however, images showed that Se^0^ nanoparticles may coalesce or aggregate to form Se^0^ spheres (**Figure 4C**). Se^0^ spheres of about ≤100 nm were less dense than their larger counterparts (**Figure 4C**).

### Photoconductivity Properties of Se^0^ Spheres

Since Se^0^ is a semi-conductor exhibiting photoconductivity properties (Qamhieh et al., 2004; Yaman et al., 2011), the conductive properties of the Se^0^ spheres produced by the granular sludge were analyzed by conductive atomic force microscopy. The results showed that these biologically produced Se^0^ spheres were non-conductive, independent of light exposure under the conditions tested (Figure S6). This is most probably due to the isolating properties of the organic material constituting the pellicle-like structures associated to the Se^0^ spheres.

### Characterization of Se^0^ Spheres by X-ray Photoelectron Spectroscopy (XPS)

**FIGURE 5:**
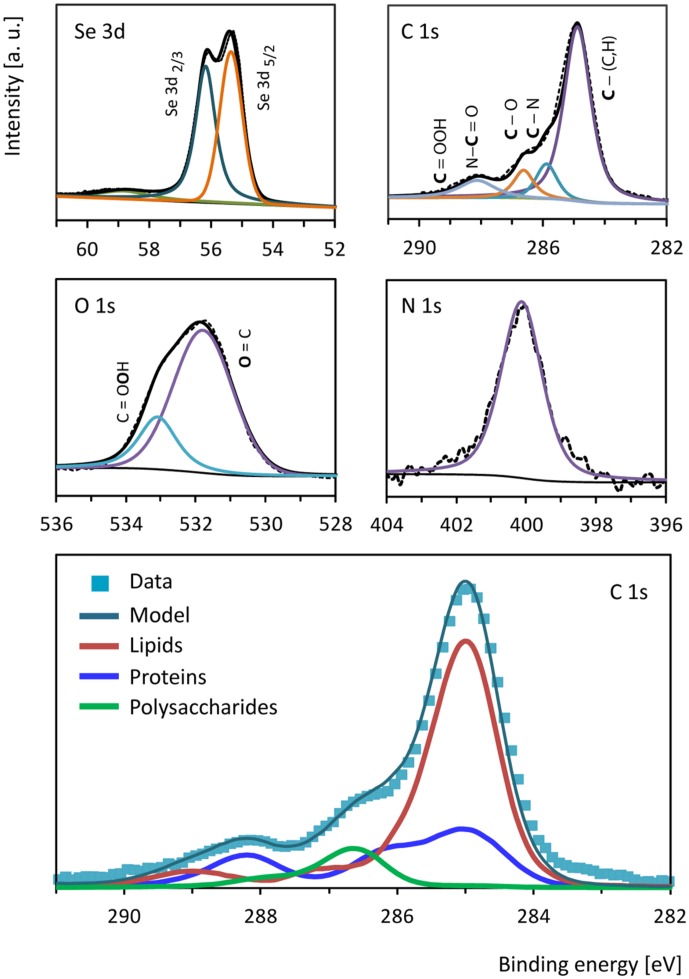
**X-ray photoelectron high resolution spectra of selenium (Se 3d), carbon (C 1s), oxygen (O 1s) and nitrogen (N 1s) from the surface of the Se^0^ spheres.** The bottom panel shows the biological components contributing to the carbon (C 1s) region as estimated based on the mathematical approach described by Ramstedt et al. (2011).

Se^0^ spheres were analyzed by XPS to investigate the nature of the surface coating or associated material. The Se^0^ spheres XPS spectrum showed signals between 54.9 and 56.3 eV (**Figure 5**) confirming the presence of elemental selenium in the spheres (Moulder et al., 1995). Besides Se^0^, the spheres contained organic material on the surface as revealed by the presence of C, N, and O (**Figure 5**). A comparison of the C 1s signal of the XPS spectrum of the microbially produced Se^0^ spheres with that of XPS spectra from microbial material and XPS spectra of various standard proteins, lipids and polysaccharides (Ramstedt et al., 2011; Rouxhet and Genet, 2011) was used to assign chemical functions to the observed peaks in the spectrum (**Figure 5**). At 285 eV, C-(C,H) bonds refer to aliphatic carbon (e.g., -CH_2_-CH_2_) of for example lipids and side chain amino acids (Ramstedt et al., 2011). At 285.9 eV, C-N bonds can be due to amines and/or amides, while at 286.6 C-O bonds were observed (e.g., as for polysaccharide material; Ramstedt et al., 2011). Carbon double bonded to oxygen as in peptide bonds (-N-C = O) was detected at 288.1 eV, while signals of the carbon peak in carboxylic acid arise at about 289 eV (Ramstedt et al., 2011). Based on a mathematical approach, which was developed to analyze the C 1s region of XPS spectra from biological samples (Ramstedt et al., 2011), the estimated fraction of proteins/peptides, lipids and polysaccharides associated to the Se^0^ spheres was 0.30, 0.61 and 0.09, respectively (**Figure 5**, bottom panel).

### Proteomic Analysis of the Organic Material Associated to the Se^0^ Spheres

**FIGURE 6:**
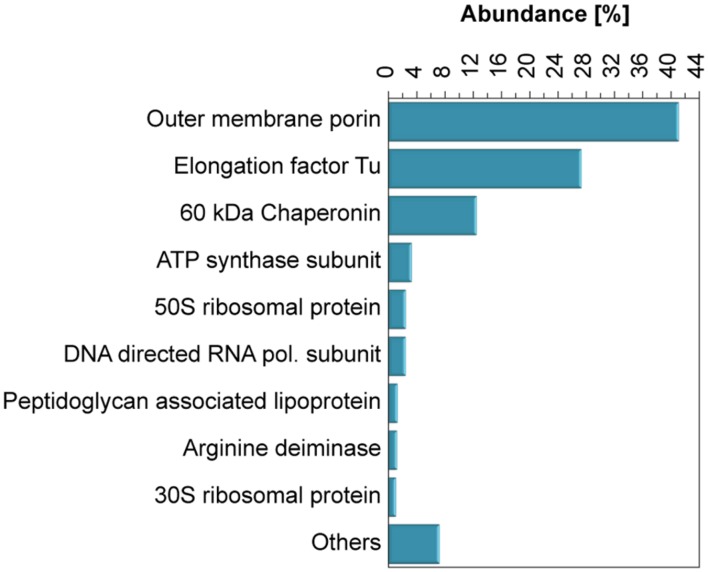
**Abundance of proteins associated to the Se^0^ spheres.** Proteins which abundance was <1% were grouped as “Others.”

A proteomic analysis of the Se^0^ spheres indicated that both proteins normally anchored to microbial membranes and proteins of cytoplasmic origin were associated to the Se^0^ spheres. The most abundant peptides matched those of outer membrane porins (40% abundance, **Figure 6**), which are trans-membrane water-filled channels with pore sizes of about 0.6–2 nm and found in the outer membrane of the dual envelope of gram-negative bacteria (Hancock, 1987). The second most abundant protein/peptides associated to the Se^0^ spheres was the elongation factor protein Tu (26% abundance, **Figure 6**), a cytoplasmic protein which facilitates translational elongation during the formation of peptide bonds on the ribosome (Aleksandrov and Field, 2013).

Other proteins found were ATP synthases and peptidoglycan lipoproteins (**Figure 6**), these are inner membrane anchored and periplasm associated proteins, respectively. In addition, proteins of ribosomal origin were found. Also of interest was the finding of arginine deaminase, a protein involved in arginine fermentation (**Figure 6**). None of the reductases specifically related to selenium respiration such as nitrite/nitrate or selenate reductases (DeMoll-Decker and Macy, 1993; Schröder et al., 1997), were detected. However, periplasmic reductases were found, namely NAD quinone oxidoreductase (0.3 %) and flavodoxin oxidoreductase (0.2 %) (Table S3).

The majority of the most abundant proteins associated to the Se^0^ spheres (**Figure 6**) were assigned to the γ-*Proteobacteria* family *Pseudomonadaceae* (Table S4; Figure S7), and specifically to the genus *Pseudomonas* (Table S4). The proteins associated to the Se^0^ spheres (**Figure 6**) strongly contrasted to a protein profile of crude extracts from the extracellular matrix of the inoculum granular sludge (i.e., not exposed to selenite, Figure S8), in which the majority of the proteins were affiliated to methanogenic archaea and γ-*Proteobacteria* (genera *Desulfovibrio*, *Geobacter*, *Synthophobacter*, and *Pelobacter*) (Table S4).

## Discussion

### Microbial Phylotypes in the Selenite Reducing Anaerobic Granules

A great variety of bacterial phylotypes reduce selenite (Stolz et al., 2006; Pearce et al., 2009). Probably the presence of a large population of γ*-Proteobacteria* and *Clostridia* contributed to the suitability of the granular sludge to carry out the selenite reduction process. In particular *Geobactereacea* (γ*-Proteobacteria*) and *Clostridiaceae* are metabolically versatile gram-negative microorganisms and species from these families reduce selenate and selenite (Pearce et al., 2009; Bao et al., 2013). qPCR results suggest that the archaeal community likely was not directly involved in selenite reduction since its abundance did not seem to change after selenite exposure (Figure S2). Methanogens are particularly sensitive to selenite which at concentrations as low as 0.05 and 0.08 mM can cause 50% loss of the hydrogenotrophic and acetoclastic methanogenic activities, respectively (Lenz et al., 2008b).

From the granular sludge used as inoculum, two main microbial phylotypes dominated the selenite reducing granular sludge: *Veillonellaceae* and *Pseudomonadaceae*. Members of the *Veillonellaceae* family, e.g., *Veillonella atypica*, are strict anaerobes and reduce selenite forming Se^0^ spheres with lactate or hydrogen as electron donor (Pearce et al., 2008). These microorganisms readily grow on lactate fermentation (Gerritse et al., 1992) and reduce selenite in a non-dissimilatory process, i.e., detoxification (Pearce et al., 2009). Their capabilities to utilize lactate and reduce selenite thus enriched *Veillonellaceae* in the selenite reducing granular sludge.

The finding of *Pseudomonadaceae* in the selenite reducing anaerobic granular sludge is of particular interest because most of the members of this family are aerobic. About 90% of the *Pseudomonadacea* of the selenite reducing granules belonged to the genus *Pseudomonas*. It is known that some *Pseudomonas* sp. are facultative and ferment arginine for anaerobic growth, and for long term survival under anaerobic conditions, these microorganisms conduct pyruvate fermentation (Schreiber et al., 2006). Most noticeably, a *Pseudomonas stutzeri* Strain pn1 was able to grow anaerobically with acetate and selenate and produced intracellular Se^0^ spheres (Narasingarao and Häggblom, 2007). *Pseudomonas* might be distinctly highly tolerant to selenite. For example, 140 mM of selenite was required to inhibit the growth of *Pseudomonas aeruginosa* by 50% (Dwivedi et al., 2013), whereas 2.4 and 0.2 mM was required to similarly inhibit *Rhodopseudomonas palustris* (Li et al., 2014a) and *Shewanella oneidensis* (Klonowska et al., 2005), respectively. Different *Pseudomonas* species grown aerobically can reduce both selenate and selenite at up to 48 mM via a detoxification process, resulting in the recovery of about 79% of the added selenite as Se^0^ (Lortie et al., 1992; Hunter and Manter, 2009).

The increased abundance of the family *Pseudomonadaceae* with the majority being *Pseudomonas* from ∼ 0.33% in the inoculum anaerobic sludge to 10% in the selenite reducing granules within a 3 weeks incubation (**Figure 1C**) correlates with the fact that members of the genus *Pseudomonas* can reduce selenate and selenite to Se^0^ spheres (Lortie et al., 1992; Hunter and Manter, 2009), they exhibit both high tolerance to selenite and have the potential to proliferate in anaerobic environments. A great majority of the proteins associated to the Se^0^ spheres affiliated to *Pseudomonas* (Figure S7; Table S3), suggesting that these microorganisms were directly involved in the reduction of selenite and the production of selenospheres.

Based on the estimated TA_P_ of the microbial community taxa in the selenite reducing granules (**Figure 1C**), it is likely that *Clostridiaceae* and *Veillionellaceae* fermented lactate, added as electron donor, while the genus *Pseudomonas* utilized the produced acetate. This study focused on the response of a mixed microbial community to selenite exposure, further studies should address in detail also the carbon metabolic fluxes of the electron donor along with the identity of phylotypes involved, e.g., via DNA/RNA-stable isotope probing.

### Multiple versus Single Intracellular Se^0^ Spheres

Multiple (**Figure 2C**) or single (**Figures 2E** and **3A**) intracellular Se^0^ spheres were produced. The occurrence of extracellular synthesis of Se^0^ particles cannot be excluded, however, most particles appeared to be intracellular and when observed extracellularly, they appeared close to damaged cells (Figure S3) suggesting their release by cell lysis. Production of intracellular spheres with subsequent expulsion is not likely based on the large size of the observed extracellular spheres. Vesicles can be produced by gram-negative bacteria in order to expulse material located in the periplasmic space. However, such vesicles are about 20–250 nm (Kulp and Kuehn, 2010) and most of the observed Se^0^ spheres (about 75%) were larger than that size (Figure S5). Thus, the majority of extracellularly observed Se^0^ spheres were most likely intracellularly produced and subsequently released following cell lysis.

### Proteins Associated to the Se^0^ Spheres Give Clues to Biosynthesis Mechanisms

This study presents the first semi-quantitative analysis of proteins associated to Se^0^ spheres which, combined with the high resolution imaging (and tomography), the high throughput sequencing of the mixed microbial community, and information on selenite bioconversion documented in the literature, allowed proposing a schematic model on how the synthesis of Se^0^ spheres might have occurred in the bacterial phylotypes (gram-negative bacteria) dominating the selenite reducing granules.

#### Proteins Associated to Se^0^ Spheres

Proteins or cell-free extracts aid in the formation of chemically synthesized spherical Se^0^ particles (Dobias et al., 2011; Zhang et al., 2012). Furthermore, proteins can restrict their size, with smaller particles observed at higher protein concentrations (e.g., of bovine serum albumin (Zhang et al., 2004, 2012). The components associated to the Se^0^ spheres thus likely contributed to guiding their synthesis and to their stability (i.e., spherical shape, mean size about 300 nm).

**FIGURE 7:**
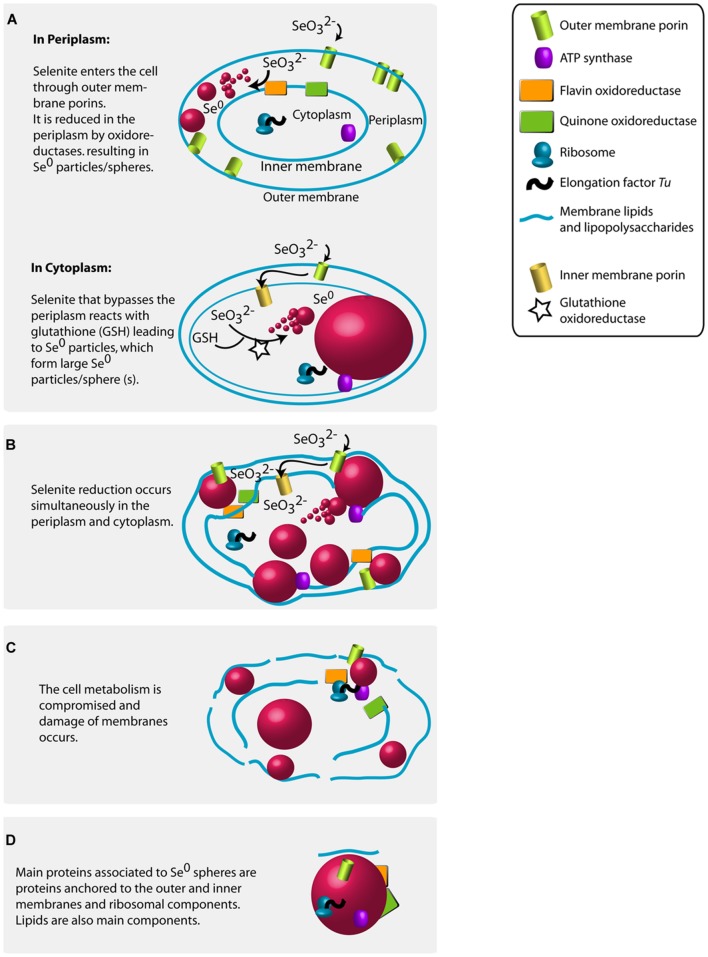
**Proposed mechanisms for the production of Se^0^ by selenite fed granular sludge bacteria based on the Se^0^ spheres proteomics, the high magnification electron microscopy imaging results of this study and previous research findings (see main text for details). (A)** Se^0^ spheres in periplasm or in cytoplasm. **(B)** Multiple Se^0^ spheres. **(C)** Damage of membranes. **(D)** Proteins associated to the produced Se^0^ spheres.

The high abundance of outer membrane porin proteins/peptides associated to the Se^0^ spheres (**Figure 6**) suggests that gram-negative bacteria were involved in their synthesis because outer membrane porins are a distinct feature of gram-negative bacteria (Silhavy et al., 2010). Selenite may pass through the outer membrane porins and may initially be reduced in the periplasm; the quinone and flavin oxidoreductases found associated to the Se^0^ spheres might have been involved in the reduction (**Figure 7A**, Table S3). Recent studies showed that periplasmic fumarate reductases from *Shewanella* and a flavin oxidoreductase of *Rhizobium selenitireducens* mediated selenite reduction (Hunter, 2014; Li et al., 2014b). The absence of glutathione reductase associated to the Se^0^ spheres does not rule out its involvement in a detoxification process, since it is possible that these proteins do not associate as strongly as the other proteins to the Se^0^ spheres or they may be inaccessible to extraction within the inner parts of the particles.

The finding of outer membrane porins and elongation factor Tu as proteins associated to Se^0^ spheres produced by the granular sludge is in accordance with the qualitative results of Dobias et al. (2011), who identified these two proteins associated to Se^0^ spheres produced by *Escherichia coli*, but did not investigate the location of particles synthesis. The finding of the elongation factor Tu as associated protein to the Se^0^ spheres is intriguing. In two independent qualitative proteomic investigations, this protein was found associated to Se^0^ spheres produced by different bacterial pure cultures (Dobias et al., 2011; Lenz et al., 2011a). In our study using mixed microbial communities in anaerobic aggregates, the semi-quantitative proteomic analysis showed that the elongation factor Tu was one of the most abundant proteins associated to the Se^0^ spheres, and most of the sequences of this protein were assigned to *Pseudomonas* (Table S3; Figure S7). Why this protein distinctively associates to the particles may be due to its amino acid composition which differs in certain aspects from the average of 108 other protein families. Specifically, the elongation factor Tu contains about 20% more charged residues than the average of other protein families (Arai et al., 1980), making it more likely to ionic interactions with the growing Se^0^ spheres. Further investigation on the interactions of this particular protein with Se^0^ is warranted since peptide sequences or motifs that selectively bind Se^0^ might be suitable capping agents to produce customized anticancer nano drugs (Wu et al., 2012; Feng et al., 2014).

### Localization of Selenite Reduction and Relation to Proteins Associated to the Se^0^ Spheres

In this study, the absolute abundance of gram-negative bacteria increased in the selenite reducing granules (**Figure 1C**). In previous reports and using pure cultures, most of the bacteria that reduce selenite to Se^0^ are phylogenetically diverse gram-negative bacteria, which cytoplasm is embedded in a double cell membrane separated from each other by a periplasmic space. Though selenite may enter the cell through a sulfate permeation system (Lindblow-Kull et al., 1995), the repression of such system does not impair selenite uptake, thus implying the existence of other selenite transport systems (Turner et al., 1998). Being outer membrane porin proteins a distinct feature of gram-negative bacteria (Silhavy et al., 2010), and that likely such protein families are linked to the transport of selenite as shown in a gram-negative bacterium (Ledgham et al., 2005), it is reasonable to envisage that selenite is transported via non-specific outer membrane porin systems, which allow the diffusion of ions (Galdiero et al., 2012). Selenite then may initially be reduced in the periplasm by reductases (Hunter, 2014; Li et al., 2014b) such as the quinone and flavin oxidoreductases (**Figure 7A**).

At high selenite concentrations, or in the absence of suitable selenite reductases in the periplasm, selenite enters the cytoplasm (**Figure 7A**). As proposed by Kessi and Hanselmann (2004), selenite readily reacts with glutathione in the cytoplasm forming selenodiglutathione, which is then converted to selenopersulfide and reduced glutathione by glutathione oxidoreductase. Selenopersulfide is unstable and transforms readily into reduced glutathione and Se^0^. Possibly, at high selenite concentrations and considering that glutathione is the most abundant thiol in some microorganisms (e.g., γ*-Proteobacteria* such as *Pseudomonas*) (Kessi and Hanselmann, 2004), the formation of Se^0^ particles in their cytoplasm may readily result in formation of a single large Se^0^ sphere (**Figures 2D** and **3A**). Due to their physical location, these Se^0^ spheres have cytoplasmic and inner membrane components associated to them such as the cytoplasmic elongation factor Tu and ribosomal proteins along with inner membrane ATP synthases as found in this study (**Figure 7A**).

Nascent (or initially precipitated) Se^0^ true nanoparticles could coalesce (Privman, 2009; Thill et al., 2012) to form large Se^0^ spheres (**Figures 4C** and **7**). Various mechanisms and mathematical models for nucleation and growth of nanoparticles of diverse elements, including silver and gold, have been proposed (e.g., coalescence, Ostwald ripening, oriented attachment) (Privman, 2009; Thill et al., 2012; Thanh et al., 2014). However, these processes have not yet been investigated in the case of selenium and dedicated kinetic studies are necessary to that end.

Periplasmic and cytoplasmic selenite reduction may not be exclusive; and can occur simultaneously in gram-negative cells (**Figure 7B**). When various foci of nascent nanoparticles occur, various spheres as observed in **Figure 2C** are produced. The high numbers of spheres occupying most of the cell volume may compromise the functioning of the cells. Single large particles may damage or cause rupture of the cell. For example, the cell damage shown in **Figure 3C** is not likely due to impact of an externally colliding Se^0^ sphere (e.g., “nano-bullet”) because the Se^0^ sphere settling velocity is very low, e.g., 3 cm day^-1^ (Buchs et al., 2013) and considering the viscoelastic resistance of bacterial cells (Vadillo-Rodriguez and Dutcher, 2009), which even allows them to pass through pores smaller than their cell size (Lebleu et al., 2009), then a plastic deformation of the cell would have been observed. Instead, a fracture was observed suggesting cell damage was initiated from inside (**Figure 3C**).

The resulting Se^0^ spheres are associated with proteins of both periplasmic and cytoplasmic origin (**Figure 7D**). Further research should investigate the lipid components as well. The XPS analysis suggests the presence of lipids on the surface of the Se^0^ spheres (**Figure 5**). These can be associated as being of membrane origin (lipids are main components of membranes (Clifton et al., 2013), but specific lipid analysis is required for detail identification (Sturt et al., 2004).

### Implications for Selenium Removal

The microbial community structure changed during the batch incubation of the anaerobic granular sludge in a medium supplemented with lactate and selenite under anaerobic conditions. An increase in the abundance of members of the family *Veillionellaceae* and the genus *Pseudomnas* occurred in the developed selenite reducing granular sludge.

Given that the Se^0^ spheres were deposited within the surface of the granular sludge and apparently mostly within the bacterial cells, it is expected that a good retention of the Se^0^ spheres can be achieved by sludge retention when the process would be run in continuous or sequencing batch reactors. Considering that multiple or single large Se^0^ spheres eventually damage or cause rupture of the cells (as observed in this study), release of such spheres is expected, thus future studies should address the time frames for recovery if continuous operation is desired to avoid sudden loses of particles as observed before (Lenz et al., 2008a).

It may be possible to selectively harvest the outer zone of selenium reducing granules through hydraulic shearing. This technique was previously applied to selectively remove various layers of anaerobic granular sludge to study the microbial communities inhabiting those layers (Lu et al., 2013). The Se^0^ spheres can then be recovered from the harvested sludge layers by inexpensive starving of the sludge material to enhance cell lysis.

The Se^0^ spheres produced in this study were capped with organic material. It is expected that the organic material associated to the Se^0^ spheres produced by the granular sludge microorganisms changes the properties of Se^0^. Organic material associated to microbially produced nanoparticles influences their transport and fate in the environment (Moreau et al., 2007; Buchs et al., 2013). Particularly, Se^0^ spheres produced by the same granular sludge used in this study have a negative zeta-potential of around -23 mV at circumneutral pH (Staicu et al., 2015), which keeps them as stable colloids and hampers their settling (Buchs et al., 2013). However, biologically produced Se^0^ spheres can be further agglomerated and settled by simple salt additions (Buchs et al., 2013). The use of marine water as a cheap source of salt solution (or medium), if available, could be used or investigated for the agglomeration of Se^0^ spheres produced by anaerobic microbial aggregates.

## Author Contributions

GG-G developed experimental design, conducted experiments, analyzed results, wrote and revised the manuscript. PL developed experimental design and revised the manuscript. PS developed experimental design and revised the manuscript.

## Conflict of Interest Statement

The authors declare that the research was conducted in the absence of any commercial or financial relationships that could be construed as a potential conflict of interest.

## Footnotes

1 http://qiime.org
