# Supplementary material for: Selenite Reduction by Anaerobic Microbial Aggregates: Microbial Community Structure, and Proteins Associated to the Produced Selenium Spheres
Source: Front Microbiol. 2016 Apr 26;7:571. doi: 10.3389/fmicb.2016.00571 (PMC4844624; doi:10.3389/fmicb.2016.00571)
Supplement: Supplementary file 1 [file Data_Sheet_1.ZIP › Supporting_information.pdf]

Selenite reduction by anaerobic microbial aggregates: Microbial community structure, and proteins associated to the produced selenium spheres

Gonzalez-Gil G., Lens PNL, Saikaly P.

SUPPORTING METHODS

Quantitative PCR primer pairs and reaction conditions

Bacterial and archaeal 16S rRNA gene fragments were amplified using primers B-341F (5′-CCTACGGGNGGCWGCAG-3′) and B-805R (5′-GACTACHVGGGTATCTAATCC-3′) for Bacteria, and A-519F (5′-CAGCMGCCGCGGTAA-3′) and A-1017R (5′GGCCATGCACCWCCTCTC-3′) for Archaea (Klindworth et al 2013 and references therein). Each 25-µl reaction contained 12.5 µl of iQ SYBR Green Super Mix (Bio-Rad Laboratories, Hercules, CA), 300 nM of each forward and reverse bacterial or archaeal primers as defined below, and 2 µl of template DNA. Cycling conditions were 3 min at 95 °C, 40 cycles of 30 s at 94 °C, 30 s at 56 °C (53 °C for archaea), and 30 s at 72 °C. After cycling, a melting curve analysis from 50 to 95 °C was conducted to verify the specificity of the reactions.

Extraction and digestion of protein fraction

The protein fraction was solubilized in a buffer containing 200 mM Tris-HCl (pH 8.0) and 0.2% (w/v) sodium dodecyl sulfate (SDS) at a 1:3 ratio, vortexed briefly and then sonicated on ice for about 5 sec with a QSonica sonicator XL2000 series (Misonix, Newton, CT) and a CML-4 probe (Misonix). Proteins were then precipitated in 10 volumes of ice-cold 10% (w/v) trichloroacetic acid (TCA) in acetone and vortexed. After a 2 h-incubation at -20°C, proteins were pelleted at 3,333 × *g* for 15 min and the solution decanted. The pellet was washed three times in 10 volumes of ice-cold 80% (v/v) acetone and collected by centrifugation at 3,333 × *g* for 10 min at 4°C after each wash. Excess acetone was air-dried, and proteins were re-suspended in 20 µL of 2 × reducing buffer (200 mM Tris-HCl (pH 6.8), 40% (v/v) glycerol, 10% (w/v) SDS, 0.02% (w/v) bromophenol blue) and reduced for 10 min at 95°C. Protein extracts were clarified by centrifugation at 13,400 × *g* for 10 min at room temperature and separated by 12% SDS-polyacrylamide gel electrophoresis (PAGE) for 1.5 h at 120 V. Gels were stained with Coomassie Brilliant Blue R-250 (Bio-Rad, Hercules, CA) and destained with Coomassie destaining solution (Bio-Rad) until an appropriate protein *versus* background ratio was obtained for visualization.

Each gel band was entirely excised, divided into four sections and each placed into a separate microcentrifuge tube. The digestion and extraction of peptides from gel pieces was conducted as described by Thomas et al. 2013.

Protein identification by LTQ-Orbitrap-Velos

A volume of 5 µL of peptide mixtures was injected onto a pre-column (5 µm, 200 Å, 50 mm long × 0.2 mm Magic C18AQ [Michrom Bioresources, Auburn, CA]). Peptides were then injected onto a separation column (3 µm, 200 Å, 150 mm long × 0.075 mm Magic C18AQ [Michrom Bioresources]). A spray voltage of 1,500 V was applied. The mobile phases consisted of 0.1% FA, 5% (v/v) ACN (buffer A) and 0.1% (v/v) FA, 90% (v/v) ACN (buffer B). A three step gradient of 0-40% buffer B in 20 min, then 40-90% buffer B in 5 min and finally 90% buffer B for 20 min with a flow of 400 nL min^{-1} over 45 min for peptide elution. The MS scan range was *m/z* 350 to 1600 and the normalized collision-induced dissociation set at 35.0 V. The top 10 precursor ions were selected in the MS scan by Orbitrap with a resolution of *r*=60,000 for fragmentation in the linear ion trap. The spray voltage, capillary voltage and capillary temperature were 1.5 kV, 47.5 V and 250°C, respectively. A sheath and auxiliary gas flow of 35 and 15 arbitrary units were used, respectively. Data were recorded using Xcalibur™ software (version 2.1; Thermo Scientific) in ".raw" format and transferred to ".mgf" using Proteome Discoverer (version 1.2.0.208; Thermo Scientific).

All spectra (".mgf" files) were submitted to a local MASCOT (version 2.4.0; Matrix Science, London, UK) server and set-up to search bacteria in the Swissprot database (release 2012), assuming the digestion enzyme trypsin, allowing up to two missed cleavages. MASCOT was searched with a parent ion tolerance of 10 ppm, a fragment ion mass tolerance of 0.5 Da. Iodoacetamide derivative of cysteine was specified as a fixed modification and oxidation of methionine residues as variable modifications. Proteins were considered positively identified if the molecular weight search (MOWSE) score from MASCOT was over the 95% confidence limit corresponding to a score ≥ 37. Data validation was performed with Scaffold (version 4.3.2; Proteome software, Portland, OR) at a protein and peptide thresholds of 95%, a minimum of one unique peptide and a protein false discovery rate (FDR) of ≤ 1%. Protein probabilities were assigned by the Protein Prophet algorithm Nesvizhskii et al 2003. Results from the two extractions were combined to increase protein coverage.

SUPPORTING TABLES

Table S1. Total number of bacterial pyrosequencing reads and alpha diversity estimates.

	Inoculum granules	Selenite reducing granules
**Total number of reads***	20887	18787
**Average read length (bp)**	424	423
**Unique OTUs (=Observed species)**	1081	817
**Chao1 (=Species richness)**	1788	1332
**Phylogenetic diversity**	93	73
**Shannon index**	7	6

* reads that passed all pipeline quality controls as specified in the materials and methods section.

Table S2. Detailed taxonomy at class, family, genus and species level of the inoculum and selenite reducing granular sludge. See excel file Supporting Table S2.

Table S3. Report of the proteomic analysis for the proteins associated to the Se^0 spheres. See excel file Supporting Table S3.

Table S4. Report for the proteomic analysis of the proteins retrieved from the extracellular matrix of the inoculum granular sludge. See excel file Supporting Table S4.

SUPPORTING FIGURES

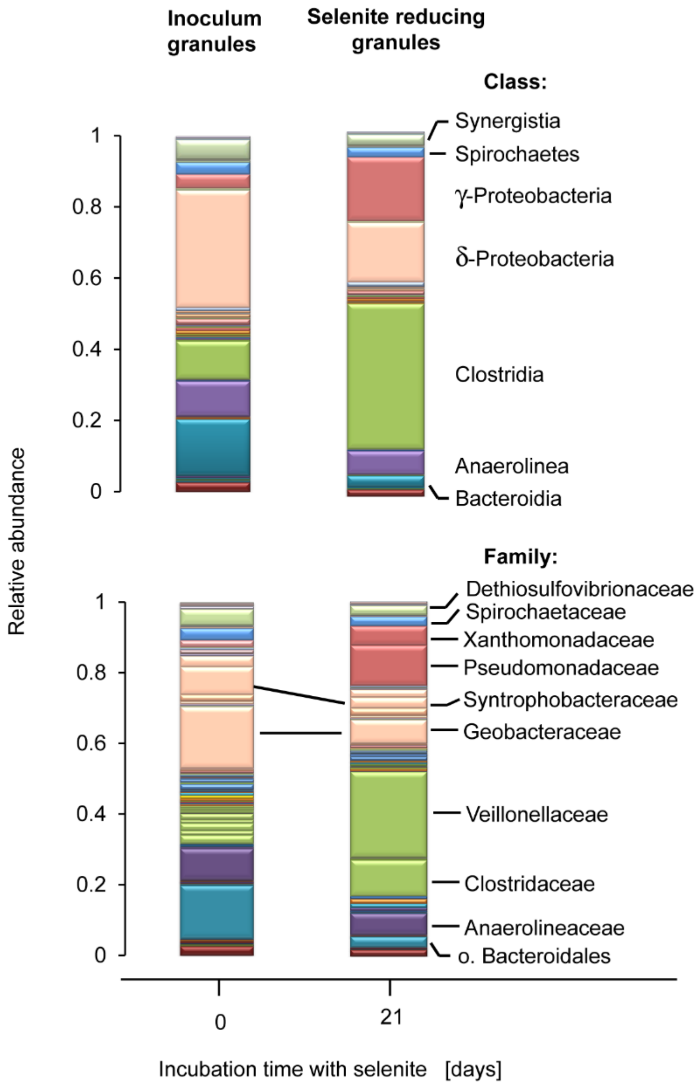

Figure S1. Bacterial community structure of the inoculum and selenite reducing granules at the class and family levels.

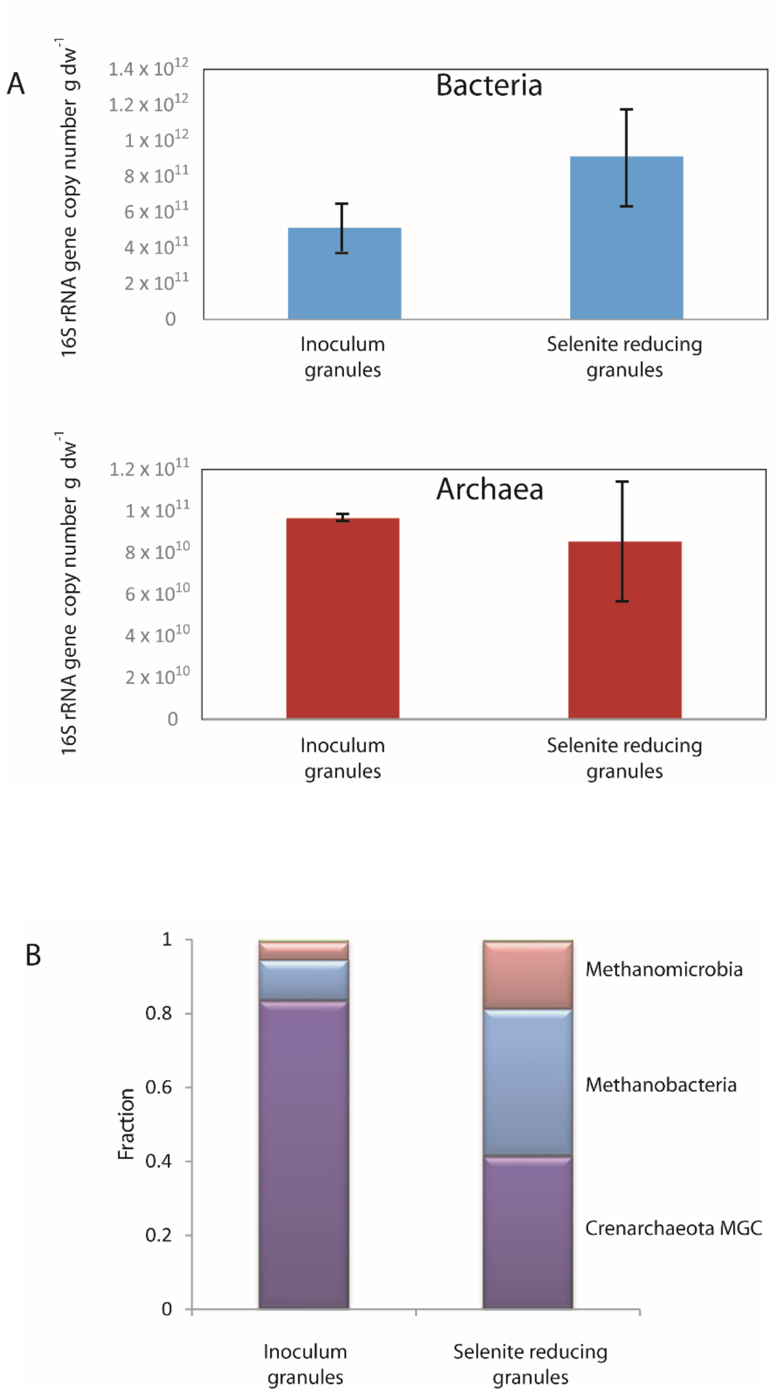

Figure S2. (A) Abundance of Bacteria and Achaea in the inoculum and in the selenite reducing granules estimated based on qPCR conducted as described in reference (3); error bars refer to standard deviation (n=3). The difference in the bacterial 16S rRNA gene copy numbers between the inoculum and selenite reducing granules was significant (*t* test, P=0.05). However, the archaeal copy numbers was not significantly different (*t* test, P=0.57). **(B)** Achaea population at the class level of the inoculum and selenite reducing granules based on pyrosequencing of archaeal 16S rRNA gene fragments analyzed as described before (Gonzalez-Gil et al 2015).

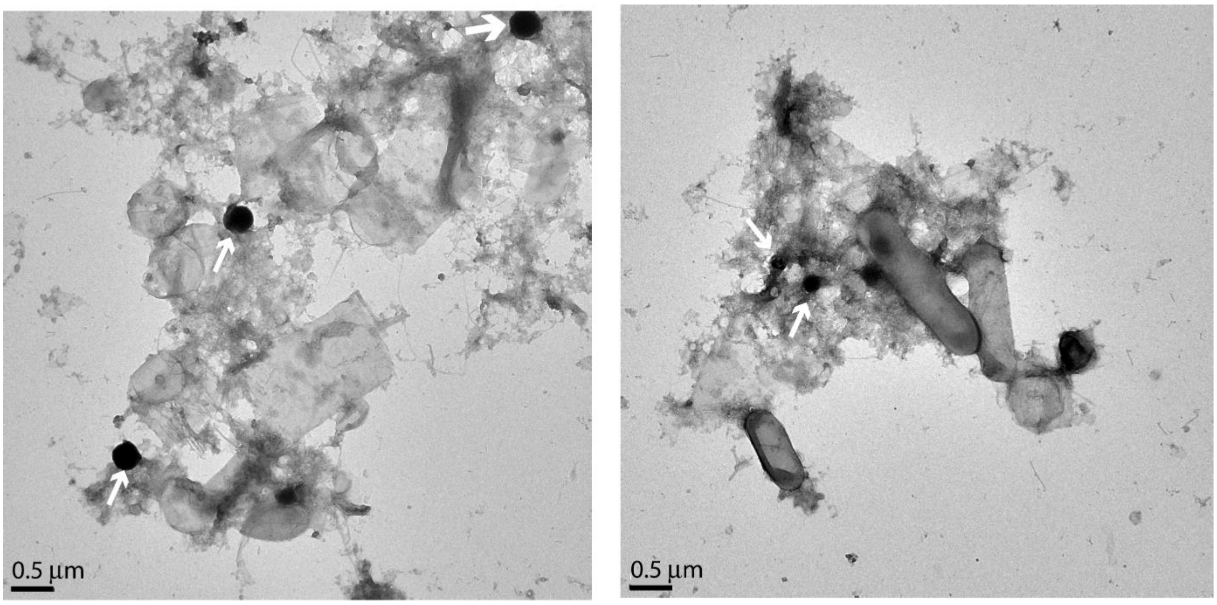

Figure S3. Transmission electron micro-images showing Se^0 spheres close to lysed cells. Some Se^0 spheres are indicated by white arrows.

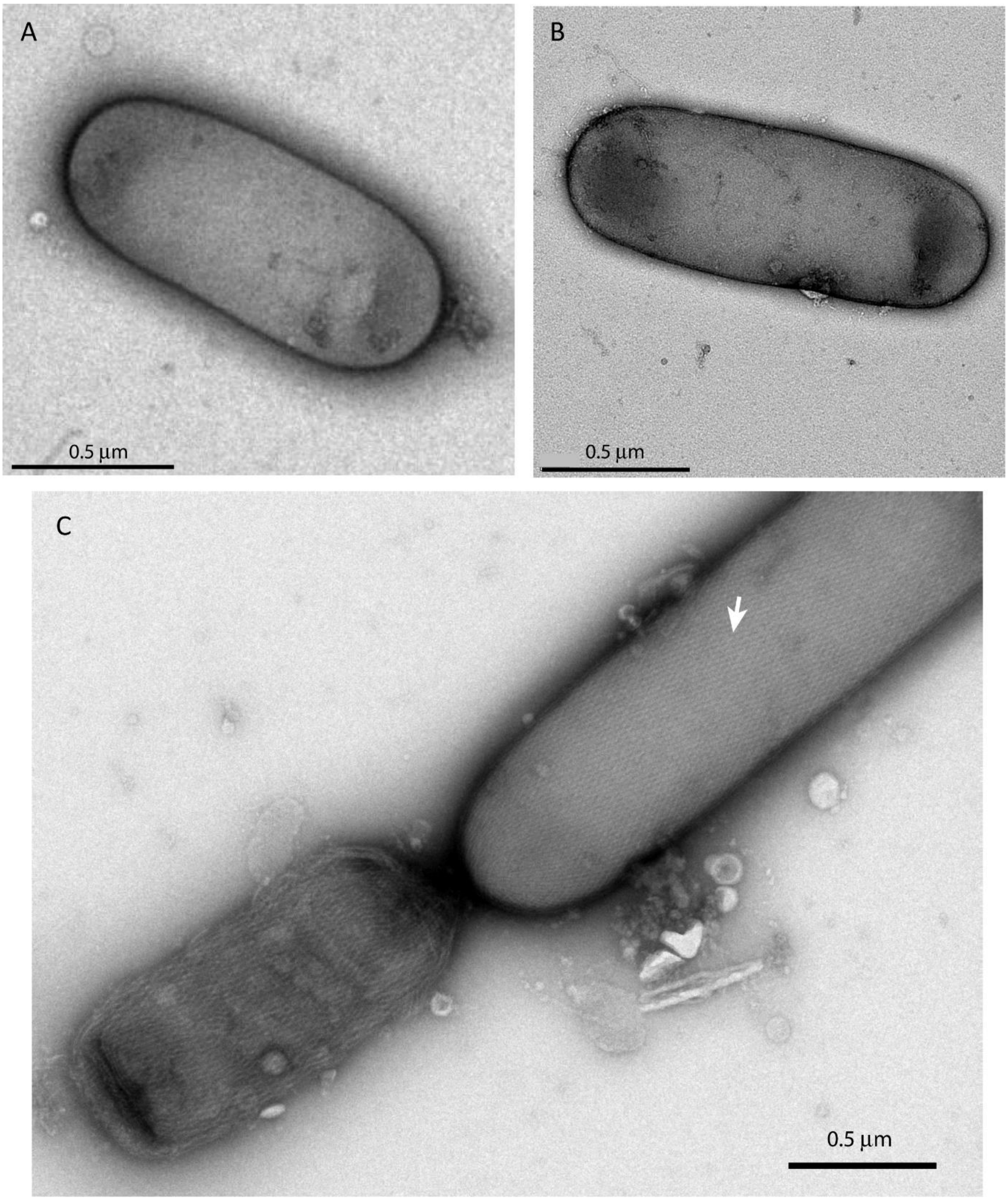

Figure S4. Transmission electron micro-images showing intact cells. The S-layer, self-assembly proteinaceous subunits, is visible on the cells from panel (C). The S-layer shows an hexagonal surface lattice (e.g., as shown by the white arrow), similar to that observed in *Clostridia* cells (Sleytr and Beveridge 1999).

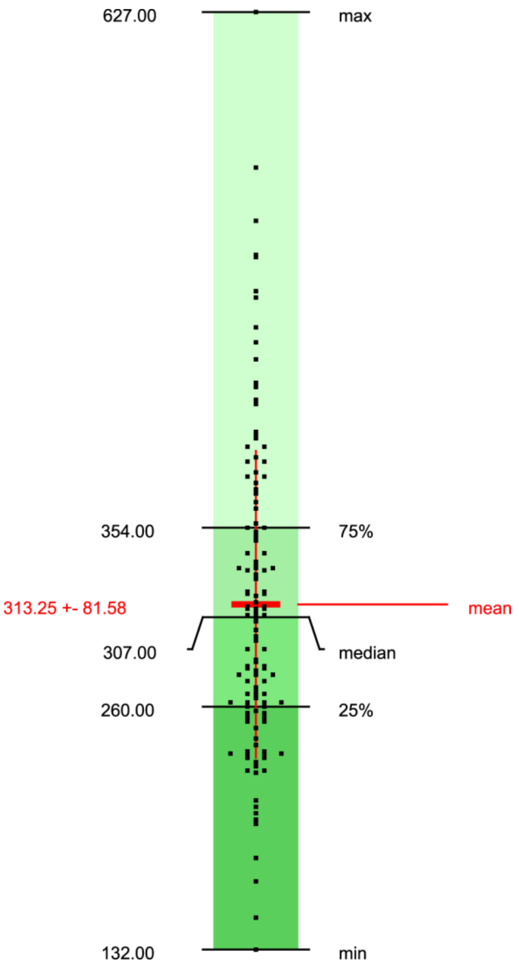

Figure S5. Size characterization of Se^0 spheres produced by the granular sludge.

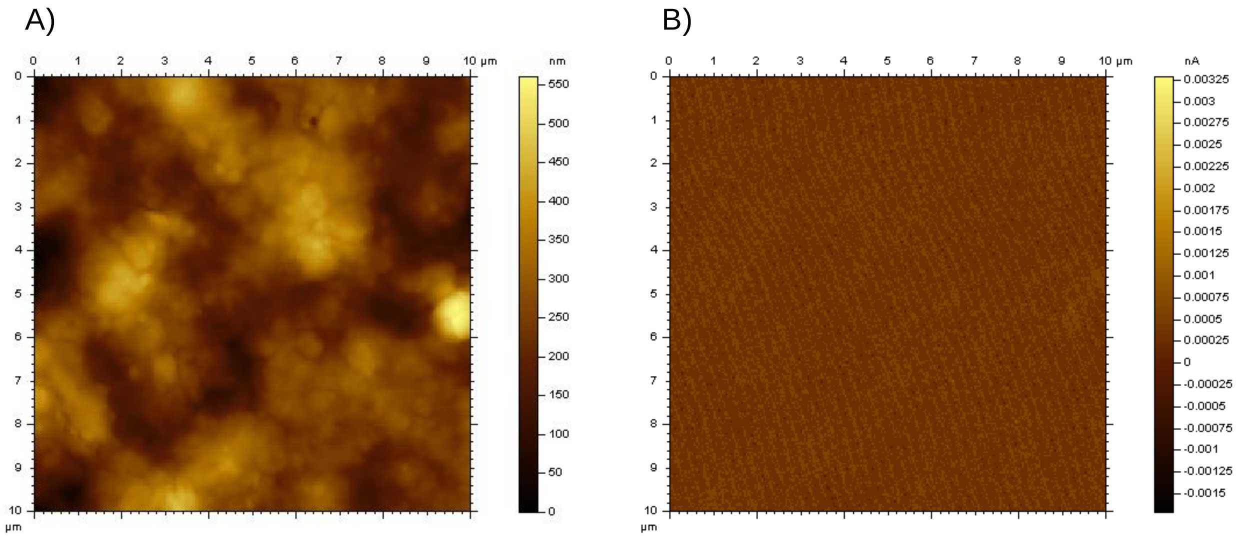

Figure S6. A) Topography and B) Conductivity of selenium nanoparticles produced by anaerobic granular sludge as measured by conductive atomic force microscopy.

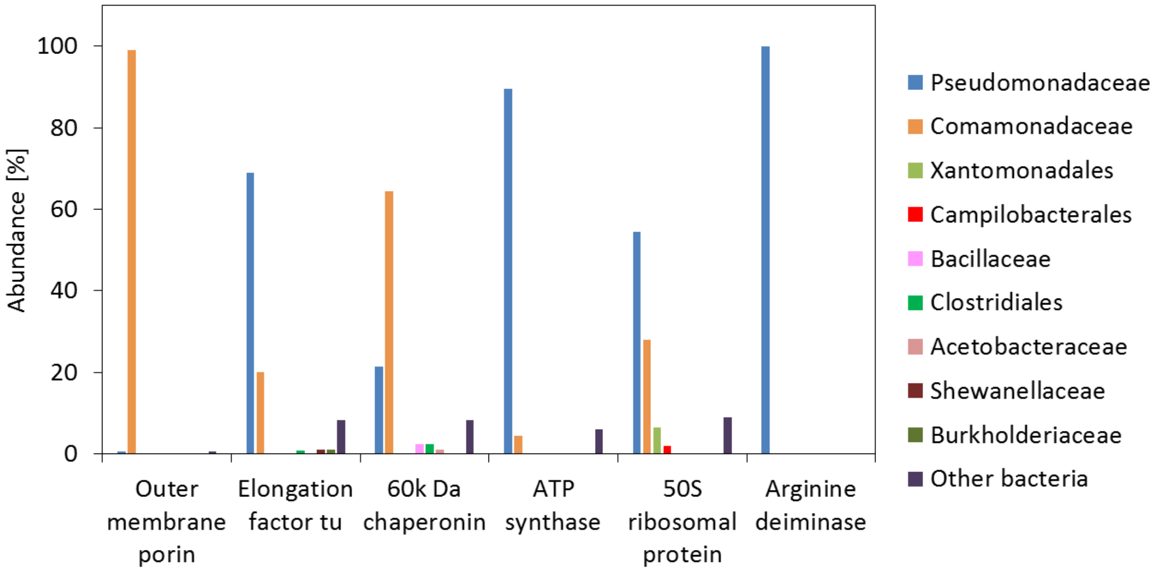

Figure S7. Taxonomic affiliation of the most abundant proteins associated to the Se^0 spheres. All *Pseudomonadacea* protein sequences were affiliated to various species of the genus *Pseudomonas* as can be seen in the supporting Table S3.

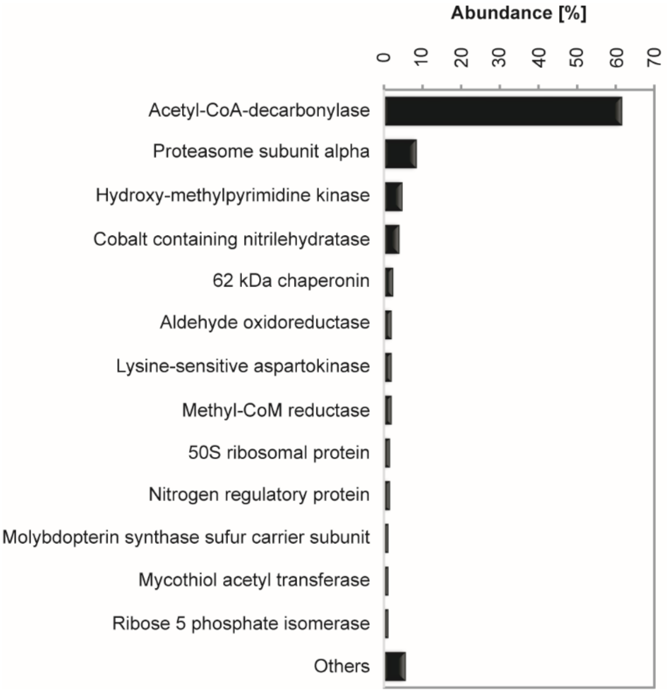

Figure S8. Abundance of proteins from a crude extract from the extracellular matrix of the inoculum granules (i.e., not exposed to selenite). Proteins which abundance was < 1 % were grouped as "Others".

References:

Gonzalez-Gil G, Sougrat R, Behzad A, Lens PL, Saikaly P. 2015. Microbial community composition and ultrastructure of granules from a full-scale anammox reactor. Microbial Ecol. **70:**118-131.

Klindworth A, Pruesse E, Schweer T, Peplies J, Quast C,HornMet al (2013) Evaluation of general 16S ribosomal RNA gene PCR primers for classical and next-generation sequencing-based diversity studies.Nucleic Acids Res **41:**e1

Nesvizhskii AI, Keller A, Kolker E, Aebersold R. 2003. A Statistical Model for Identifying Proteins by Tandem Mass Spectrometry. Anal. Chem. **75:**4646-4658.

Sleytr UB, Beveridge TJ. 1999. Bacterial S-layers. Trends Microbiol. **7:**253-260.

Thomas L, Marondedze C, Ederli L, Pasqualini S, Gehring C. 2013. Proteomic signatures implicate cAMP in light and temperature responses in Arabidopsis thaliana. Journal of Proteomics **83:**47-59
